# Use and Abuse of Entropy in Biology: A Case for Caliber

**DOI:** 10.3390/e22121335

**Published:** 2020-11-25

**Authors:** Ty N. F. Roach

**Affiliations:** Hawaiʻi Institute of Marine Biology, University of Hawaiʻi at Mānoa, Kāneʻohe, HI 96744, USA; smokinroachjr@gmail.com

**Keywords:** biophysics, biochemistry, dynamical systems, diversity, complexity, path information

## Abstract

Here, I discuss entropy and its use as a tool in fields of biology such as bioenergetics, ecology, and evolutionary biology. Statistical entropy concepts including Shannon’s diversity, configurational entropy, and informational entropy are discussed in connection to their use in describing the diversity, heterogeneity, and spatial patterning of biological systems. The use of entropy as a measure of biological complexity is also discussed, and I explore the extension of thermodynamic entropy principles to open, nonequilibrium systems operating in finite time. I conclude with suggestions for use of caliber, a metric similar to entropy but for time-dependent trajectories rather than static distributions, and propose the complementary notion of path information.

## 1. Introduction

Entropy principles have been used to describe biological patterns and processes at a range of scales [1]. Perhaps the most well-known use of entropy in biology stems from the use of Shannon’s entropy (*H*) [2] to describe the diversity of an ecological community. Entropy has also been used in ecology to describe spatial patterning [3] and interconnectedness of organisms in systems [4]. In evolutionary biology, entropy principles have been used to describe the irreversible change of systems through time [5] and to quantify the organization and complexity of populations and communities [6,7]. Other uses include quantifying the thermal efficiency of organismal metabolism [8,9] and creating orientors for in silico models [10,11]. Herein, I review the general uses and misuses of entropy methods in biology and discuss other, more process-focused methods such as caliber and path information.

## 2. Uses of Entropy in Biology

In classical thermodynamics, entropy (*S*) is an extensive state variable (i.e., a state variable that changes proportionally as the size of the system changes, and is thus additive for subsystems,) which describes the relationship between the heat flow (δQ) and the temperature (*T*) of a system. Mathematically denoted, the relationship is dS=δQ/T. This formalism of entropy and Clausius’s statement of the second law of thermodynamics led to the interpretation of entropy as a measure of unavailability (i.e., entropy as a measure of the energy dispersed as heat, which cannot perform work at a given temperature). It is also this formalism which has allowed for entropy production as a measure of spontaneity, unidirectionality, and dissipation. This formalism has proven particularly useful in biology for measuring the energy dissipation and thermodynamic efficiency in biological systems including cells, organisms, and ecosystems [8,9,12,13].

The direct relationship of entropy to temperature and heat allows for the precise calculations of entropy production in systems via calorimetry and spectroscopy. These methods have proven quite valuable as a means to collect data on energetics and entropy production in biological systems, and improvements in resolution and accuracy in both technologies continue to advance bioenergetics research.

### 2.1. Statistical Entropy

The thermodynamic entropy function proposed by Clausius was extended to the field of statistical mechanics by Boltzmann with the introduction of statistical entropy [14]. In Boltzmann’s formalism, entropy is a measure of the number of possible microscopic states (or microstates) of a system in thermodynamic equilibrium, consistent with its macroscopic thermodynamic properties (or macrostate). Thus, the popular expression of entropy as $S=k_{B}ln\Omega$, where Ω is the number of microstates consistent with the equilibrium macrostate and $k_{B}$ is a constant, which serves to keep entropy in the units of heat capacity (i.e., Joules∙Kelvin^−1^). Gibbs extended Boltzmann’s analysis of a single multiparticle system to the analysis of an ensemble of infinitely many copies of the same system, demonstrating that the entropy of a system is related to the probability of being in a given microstate during the system’s fluctuations (*p_i_*), and resulting in the well-known Gibbs entropy equation:

(1) $$S=-k_{B}\underset{i}{\sum }p_{i}\ln p_{i}$$

It is notable that the Gibbs entropy less the Boltzmann constant is identical to the Shannon entropy (*H*) where

(2) $$H=-\overset{n}{\underset{i=1}{\sum }}p_{i}\log p_{i}$$

As the Gibbs entropy approaches the Clausius entropy in the thermodynamic limit, this interesting link between Shannon’s entropy and thermodynamic entropy has often led to misinterpretations of the second law of thermodynamics in biological systems (e.g., the postulation of macroscopic second laws acting at the scale of organisms and ecosystems). However, it is this same link that has made possible the idea of information engines (e.g., [15,16]) and has allowed for use of entropy concepts in many systems far removed from the heat engine (e.g., chemical systems, electrical systems, biological systems).

In biology, perhaps the most well-known application of entropy is the use of Shannon’s entropy as a measure of diversity [17,18]. More precisely, the Shannon entropy of a biological community describes the distribution of individuals (these could be individual biomolecules, genes, cells, organism, or populations) into distinct states (these states could be different types of molecules, types of cells, species of organism, etc.). The Shannon entropy normalized by the richness (i.e., the number of states) yields another diversity metric known as evenness [19,20,21], which is typically interpreted as a measure of how similar the abundances of different states are.

Beyond allowing for the calculation of diversity, entropy concepts have also been quite useful as a metric to quantify the organization, complexity, and order of biological systems. Often, this is accomplished by comparing the entropy of the system to the system’s maximum entropy (i.e., the entropy of the system without the informational constraints of history) to estimate its departure from maximum homogeneity and randomness [7]. By extending entropy-based biodiversity and complexity measures into spatially explicit landscapes, the field of landscape ecology has made particular use of entropy methods to describe spatial and topological patterning at different scales. Recent advances in the field have made use of more generalized statistical entropy formulations such as Renyi’s entropy [22] and generalized Boltzmann entropy for landscape mosaics and landscape gradients [23,24]. See *Entropy* Special Issue: *Entropy in Landscape Ecology* for other uses of entropy in this field [25].

### 2.2. Information

In the post-Shannon age, information (*I*) has been conceptualized as a form of negative entropy—that is to say that entropy is the information missing about a system, which would allow that same system to do work at a given temperature. To state it more explicitly, I=−S. This relationship has allowed for many interesting applications of entropy methods to the informational content of biological systems (e.g., informational content in a single biomolecule, a genome, a metagenome). Several biologists have applied these information theoretic approaches to model ecological and evolutionary systems. Of particular note are the infodynamic formalisms proposed by Jeff Wicken [26,27,28], Stanley Salthe [29,30,31], and Robert Ulanowicz’s concept of *ascendency* for the development and succession of ecological systems [32]. 

## 3. Abuse of Entropy in Biology

### 3.1. Entropy and Order

Despite the numerous uses of entropy concepts in biology, there has also been some confusion concerning entropy and its applications in the life sciences. One such issue is the interpretation of entropy as the disorder of a system. While entropy has often been taught in college chemistry and physics classes as synonymous with disorder, this is not actually the case. In fact, in many systems, order increases as a direct result of increasing entropy (e.g., [7]). This is because both order and disorder are functions of entropy [33]. The mathematical relationship between entropy (*S*) and disorder (*D*) is $D=S/S_{max}$. This leads to the complementary notion of order (*O*), where $O=1-S/S_{max}$, where *S_max_* is the maximum entropy (i.e., the entropy the system would have if it were free of informational constraints). The misinterpretation of entropy as disorder has led some authors to assert that the increase in biological order observed through time in many systems is a violation of the second law of thermodynamics, which is simply wrong. This relationship between entropy and maximum entropy has been useful in the areas of complexity science and autopoiesis (the study of systems capable of maintaining and reproducing themselves) [34,35]. It should be noted that the relationship of entropy to notions of order, organization, and complexity all transform the extensivity of entropy into an intensive quantity by normalizing to some other variable. This emphasizes that these metrics, although derived from entropy, are not synonymous with entropy itself.

### 3.2. Entropy-Driven Systems

Another abuse of entropy in biology is the claim that biological systems are driven by entropy (or entropy production). This notion may have begun with Schrödinger’s statement that life feeds off negative entropy [36]. However, just because entropy increases in spontaneous processes does not mean that entropy (or its production) is the ultimate thermodynamic driving force. In fact, only in the case of isolated systems does entropy alone determine the direction of thermodynamic equilibrium. For non-isolated systems such as biological systems, where there are flows of matter, energy, and entropy into and out of the systems, the movement of the system toward equilibrium is determined by both the maximization of entropy and the minimization of free energy. Only in isolated systems where internal energy (*U*) is held constant will entropy reach its maximum [37]. In the thermodynamic limit where systems undergo isentropic change (i.e., they change without production of entropy), equilibrium is only determined by the minimization of free energy. Thus, it is seen that non-isolated systems are driven by free energy flux or, more precisely, exergy flux. (Note: For those not familiar with exergy, it is the work that could be extracted in a process that reversibly brings a system to equilibrium with the environment. At constant environmental temperature and pressure, exergy change is equal to the change in free energy.) It is noted that biological systems increase the global entropy and dissipation by using free energy to create local entropy minima (i.e., building up local information and order). However, this increase in universal entropy (Δ*S^U^*) is not the driving force in biological processes but is merely a requisite of the system operating in finite time. Thus, for biological systems, just as for heat engines, the entropy production is simply a byproduct due to dissipative processes such as friction and turbulence, which should be minimized insofar as the constraints of finite time and resources allow. Consequently, it is seen that biological systems are not selected to maximize dissipation and entropy production, as is often claimed (e.g., [38]), but rather to minimize these quantities to perform work to ultimately survive and reproduce.

### 3.3. Mischaracterization of Biological Systems

Much of the confusion concerning the entropy-driven nature of biological systems stems from the mischaracterization of biological systems as isolated or closed systems (i.e., a system with no exchange of matter and/or energy). However, biological systems are not closed systems; rather, they are open to the exchange of both matter and energy. This means that they can create and maintain localized decreases in entropy by using exogenous sources of free energy and matter. It is by maintaining their localized entropy at lower levels than the surrounding environment that biological systems are able to build up information and order. This highlights the fact that biological systems must dissipate entropy into the surrounding environment to build up local order. Incorporating the role of the environment into thermodynamic studies emphasizes the ecological nature (i.e., the interconnectedness of multiple systems and subsystems to each other and to their physical surroundings) of biological systems and allows for a more complete systems view of biology.

Similar to the mischaracterization of biological systems as closed systems is the mischaracterization of biological systems as being at equilibrium. Almost by definition, living systems are not at equilibrium. Of course, some degrees of freedom may be at or near equilibrium; however, many degrees of freedom are actually quite far from thermodynamic equilibrium. The formalization of nonequilibrium approaches to open thermodynamic systems has allowed for better use of entropy principles in biology [39,40,41,42]. However, thermodynamic entropy (e.g., Clausius, Gibbs, and Boltzmann) is undefined in nonequilibrium states, as there is no well-defined temperature. This can be overcome with the use of von Neumann entropy, an extension of classical entropy to quantum mechanics. The von Neumann entropy can be calculated for any quantum state, equilibrium or otherwise, as S=−tr(ρ lnρ), where *ρ* is the density matrix describing the quantum state and *tr* is the trace of this matrix. Although von Neumann’s formulation allows for the calculation of nonequilibrium entropy, it is still a state function. Thus, one can only infer the change in entropy in a system by comparing the difference in entropy between states. This serves to highlight that thermodynamics is not really dynamics at all; rather, it is a form of comparative statics (i.e., comparing the difference between states). However, many biologists are not interested in merely comparing states, but instead aim to understand the underlying dynamics of biological processes.

## 4. Caliber

To begin to shift biological thermodynamics from a state-focused form of comparative statics to process-focused dynamics, I propose the use of caliber concepts and methodologies. *Caliber* (*C*), also known as path entropy (a notion similar to Feynman’s path integral formulation [43]), is a thermodynamic quantity that defines the distribution of flows over pathways in dynamical processes. Mathematically, this amounts to an entropy-like equation: $C=-\underset{i}{\sum }p_{i}lnp_{i}$, where the *p_i_*s here are the relative populations of flow paths [44]. This is contrary to the formulation of entropy where the *p_i_*s are relative populations of states, whereas for caliber the probabilities represent the distributions of dynamical trajectories between states. Thus, caliber is to dynamics as entropy is to comparative statics. Another way to say this is that while entropy provides a state-focused, equilibrium approach to problems, caliber provides a process-focused, nonequilibrium approach. As biological systems are inherently nonequilibrium dynamical systems, caliber provides a function, which may be better suited to accurately describe the processes occurring in cells, organisms, populations and ecosystems.

Maximum caliber approaches have been used to accurately predict dynamical distribution functions that characterize the relative probabilities of different microtrajectories, including so-called “bad actors” that contribute net motion in the direction opposite to the macroflux predicted by the second law of thermodynamics [45]. Furthermore, caliber approaches have been shown to be particularly useful in systems with a small number of individuals, where maximum caliber methods have been used to successfully model autoactivation in single-gene circuits [46]. Caliber approaches have also been shown to work well in systems involving feedback mechanisms, such as the feedback produced by the changing fitness landscape topologies arising from ecological interactions in evolutionary systems [47]. In addition to being a more informative and accurate method for predicting trajectories in changing biological systems, maximum caliber methodologies are also more parsimonious than other methods involving master equations and mass action laws, as maximum caliber requires fewer model assumptions and parameters. Thus, generalizable stochastic models utilizing caliber may be the best approach to model biological processes, such as evolution and succession, based on thermodynamic principles. For more on caliber methods and their use in biology, see the review by Ghosh et al. [48]

## 5. Discussion

### 5.1. Connecting Caliber to Other Thermodynamic Quantities

Despite the seeming advantages of caliber approaches over entropy to describe nonequilibrium dynamical systems, there are still some advantages of using entropy for certain problems. One of the major advantages to entropy methods is the strong fundamental connection between entropy and other thermodynamic metrics such as heat, free energy, work, and efficiency. The relationship between these metrics and caliber is less well-defined in the existing literature and offers an area rich for further research.

### 5.2. Informed Pathways

The link between information and entropy demonstrates that local decreases in entropy essentially amount to local increases in information. These local accumulations of information represent constraints on specific degrees of freedom which allow for control of a system as it relaxes toward equilibrium. As the approach to equilibrium is inherently a dynamical process, I propose a complementary notion to caliber which I call *path information*. Path information serves to quantify the informational constraints, which limit the possible flow paths, and ultimately allow a system to extract work from flows of free energy through these informed pathways.

This is precisely how many biological systems function: they use free energy to locally decrease entropy (i.e., create informational constraints on the flow of free energy through the system) in order to perform work. In the case of organisms, these informational constraints include the information encoded in DNA sequences and complex biochemical modifications (e.g., methylation, ubiquitination, phosphorylation). At the biochemical level, these informed pathways can be seen in the form of enzymes and molecular motors which carefully control biochemical processes. It should be noted here that the flow of free energy through informed pathways to do work does not necessarily lead to entropy production; instead, it is the thermalization of free energy which causes entropy production. This highlights a major difference between the biological mechanism of energy use and many other technologies, such as the heat engine. Rather than converting chemical energy—“food”—to heat and then using that heat to do work as it flows to a cooler subsystem, biological pathways extract work in a nonthermal manner by carefully choregraphing molecular motions to better maximize their efficiency. The buildup of path information is what allows biology to extract work from flows of free energy in this manner and ultimately what determines the thermodynamic efficiency of biological processes. This emphasizes the fact that the efficiency of living systems—that is, the degree to which they can approach the thermodynamic limits—is a matter of engineering-informed energy flux pathways, not a matter of the available free energy quantity or quality.

The formulation of path information, much like entropy and caliber, has the potential to be used in both a physical, thermodynamic sense and in a macroscopic, descriptive manner. For example, path information can be used to define the relative flows of molecules through specific biochemical pathways and quantify how this relates to metabolic efficiency. At the macroscopic scale, path information could be useful to describe dynamical processes such as organismal migration, epidemiological spread of pathogens, evolutionary gene flow, and trophic cascades. Other areas where path information formulations may prove particularly useful include hydrology, hydrodynamics, aerodynamics, and economics.

### 5.3. Ergodicity

Many authors have asserted that biological systems are ergodic (e.g., [49,50,51]). A system is ergodic if its dynamics sample phase space such that, in the long run, time averages over a single trajectory and ensemble averages over many independent trajectories yield the same result. Thus, it follows that in ergodic systems all accessible microstates are equiprobable over a long period of time and that long-term behaviors are essentially independent of initial conditions. However, this is most certainly not a characteristic of biological systems, which typically have strong signatures of history and initial conditions [52]. Furthermore, one cannot exchange space and time averages in biological systems. For one illustrative example at the microscopic scale, consider a circular, unidirectional process such as A→B→C→A in steady state. Many biochemical processes are exactly of this circular type—e.g., the buildup and breakdown of glycogen. Since this cycling of glycogen costs ATP for every cycle, there is no energetic equiprobability among the states, even though their numbers (concentrations) may remain constant. Thus, these systems are not an equilibrium ensemble and are not ergodic. This becomes even more blatantly obvious at the macroscale where ecological and evolutionary systems typically evolve to different forms over long time periods. Take, for example, the death of an organism. Once the organism has died, there is no chance of it spontaneously returning to the previous state of being alive. It should be noted here that, despite the claim of some authors, death is not synonymous with reaching equilibrium; although the organism’s metabolism has stopped, the macromolecules contained in the biomass still have a relatively high energetic potential and will continue to be oxidized to lower and lower energetic states before being recycled and assimilated into new forms. Throughout all the stages of life, death, and decomposition, there is extraordinarily little chance of spontaneously returning to the previous phase space. The non-ergodicity of biological systems is even more conspicuous in the process of mass death, or extinction, in which whole groups of organisms are lost, and although similar organisms may eventually evolve, the return to the previous biological phase space is nearly infinitesimal (especially without energetic inputs). Therefore, it is noted that biological systems are strongly constrained by history and the arrow of time, which by definition makes them non-ergodic.

Some subset of ecological and evolutionary systems may explore only a small portion of their phase space over relatively short timescales, and in this sense may have local ergodic periods characterized by macroscopic stationary states. However, over long timescales, these systems typically evolve to search a new, small area of phase space. Thus, it is seen that biological systems at all scales are largely non-ergodic.

## 6. Conclusions

The applications of thermodynamics to biological systems have been largely focused on state functions (e.g., entropy). However, this approach does not allow for the observation of process dynamics that occur in biological systems. Thus, the dynamics are only inferred from comparative statics. To move forward, the field needs to shift from a state-based science to a process-based discipline. This will necessitate the explicit incorporation of time and rate dependency, which will require the integration of other branches of physics such as statistical mechanics and kinetics. Furthermore, the type of biological data collected will need to explicitly include time as a variable. Although some authors approach biological systems as ergodic [49,50,51], allowing for the exchange of space averages for time averages or vice versa, living systems are not actually ergodic. This means that temporally resolved data is needed to address path dependency and process functions. Utilizing nonequilibrium, process-focused metrics such as caliber and path information will allow better modeling of biological processes and will give biologists a more complete and realistic understanding of the dynamics of living systems.
